# Use of Citrus Peel Waste as Bio-Fillers in Polyester Resin Composites: Analysis of Mechanical Properties

**DOI:** 10.3390/ma19040705

**Published:** 2026-02-12

**Authors:** Mariola Jureczko, Małgorzata Dziekońska, Tomasz Czapla, Bożena Gzik-Zroska, Kamil Joszko

**Affiliations:** 1 Department of Theoretical and Applied Mechanics, Faculty of Mechanical Engineering, Silesian University of Technology, Konarskiego 18A, 44-100 Gliwice, Poland; tczapla@polsl.pl; 2 Department of Engineering Materials and Biomaterials, Faculty of Mechanical Engineering, Silesian University of Technology, Konarskiego 18A, 44-100 Gliwice, Poland; 3 Department of Biomechatronics, Faculty of Biomedical Engineering, Silesian University of Technology, ul. Roosevelta 40, 41-800 Zabrze, Poland; bozena.gzik-zroska@polsl.pl (B.G.-Z.);

**Keywords:** bio-fillers, citrus peel waste, polyester resin composites, mechanical properties, circular economy

## Abstract

In the context of global trends in sustainability and the circular economy (CE), this article aims to investigate the potential of microparticles derived from citrus peel waste (grapefruit, key lime, lemon, and orange), constituting approximately 50% of the fruit weight, as eco-friendly bio-fillers in polymer composites, thereby reducing the consumption of petrochemical resins. The composites were fabricated by gravity casting using polyester resin (PR) as the matrix at filler concentrations of 2.5%, 5%, and 10% by weight. Functional properties were assessed using static tensile testing (measuring Peak Load, Peak Stress, and Young’s modulus) and Shore D hardness testing. The incorporation of unprocessed fillers generally decreased tensile strength (Peak Stress REF: 31.48 MPa), attributed to poor interfacial adhesion. The lowest Peak Stress value was recorded for the 2.5O composite (16.04 MPa). The exception was the 10K composite (10 wt.%key limee), which achieved a Peak Load (1.28 kN) nearly identical to the neat resin (1.29 kN), although the Peak Stress remained lower due to the reduced effective cross-sectional area. Stiffness (Young’s modulus REF: 3.26 GPa) increased by more than 10 wt.% for 5G (3.63 GPa), indicating effective reinforcement at this concentration. A key positive finding was a universal increase in Shore D hardness across all biocomposites (REF: 78.4 ShD), with a maximum of 83.8 ShD for 10L (lemon), a typical response to rigid fillers that suggests enhanced surface resistance. The results suggest that citrus peel waste could be considered for non-structural applications where surface durability and efficient waste management are priorities.

## 1. Introduction

Modern materials engineering is characterised by a growing interest in composite materials, primarily driven by their unique combination of desirable properties—including low density—and the simplicity and cost-effectiveness of their manufacturing processes [1,2]. However, it should be emphasised that not all composites are characterised by simple or inexpensive production processes. For example, the production of carbon fibre composites for the aerospace industry is expensive and complex. However, in this case, the resulting material properties compensate for this [3,4]. Global research in materials science focuses on continuously improving the performance properties of this class of materials. A key strategy in this context is the incorporation of various types of reinforcements, such as fibres [5,6,7]. These efforts aim to optimise the mechanical, thermal, and other functional properties of polymer composites. The incorporation of reinforcement usually results in a composite that is more advantageous than the neat polymer, owing to its lower density, higher strength, greater stiffness, and enhanced wear resistance [8,9,10,11]. However, in some cases, this results in reduced impact resistance or increased brittleness. Additionally, fibre-reinforced polymer composites also have significant drawbacks. For example, synthetic fibre composites are generally not biodegradable, and some of them (e.g., glass, aramid, and ceramic fibres) may pose potential respiratory health risks during manufacturing. An effective environmental protection strategy may be to replace synthetic fillers with natural ones.

Polymer composites reinforced with fibres of organic origin exhibit a favourable combination of mechanical and environmental properties. They exhibit satisfactory mechanical strength, low density, high specific modulus of elasticity, low coefficient of friction, and good wear and erosion resistance [12,13,14]. Additionally, owing to the biodegradability of the organic phase and the potential for cost-effective post-use waste management, these materials are an attractive alternative to composites reinforced with synthetic fibres, and are consistent with growing environmental awareness and sustainable development principles. Consequently, there has been a dynamic rise in research on so-called biocomposites, the design and manufacturing of which align with current sustainable development trends, including minimising environmental impact, rational resource management, and the production of recyclable products [15,16,17,18]. Furthermore, research on biocomposites supports the circular economy (CE) strategy promoted by the European Commission, which aims to minimise waste generation, encourage reuse, and increase resource efficiency [19,20]. Various types of industrial, construction, and biological waste can be effectively used to produce fillers for biocomposites, as described in numerous studies [21,22,23,24,25]. Agricultural waste, such as that generated during livestock farming, is also used as a filler for biocomposites [26,27,28].

An exciting direction in the development of biocomposites is the use of biological raw materials, such as plant residues, which constitute readily accessible, renewable sources. Numerous examples of the use of plant-derived components, including stems, leaves, and seed shells, have been reported in the literature. It has been shown that shells of walnuts, hazelnuts, and sunflower seeds can effectively enhance the stiffness and strength of epoxy resins, serving as an environmentally friendly alternative to conventional mineral fillers [29,30,31]. Furthermore, orange processing waste has been shown to be a potential filler that improves both the mechanical strength and sliding wear resistance of polymer composites [32,33,34]. The literature also highlights the use of fibres derived from pineapple leaves and peanut shells for insulation applications, further confirming the broad potential of biomaterials in modern materials technologies [35,36]. Several studies have shown that citrus fruit processing waste, when used as bio-fillers in biocomposites, can improve their mechanical properties and enhance their sliding wear resistance. Due to the presence of plant fibres, pectins, and polysaccharides, these materials have the potential to beneficially influence the internal structure, mechanical strength, and sorption properties of composites. Hitesh Sharma and colleagues made a significant contribution to the literature by researching the use of citrus peels as bio-fillers in epoxy composites. In their 2019 study, the authors evaluated the effect of adding lemon peel (Citrus Lemon Peel, CLP) on the mechanical properties of epoxy composites, demonstrating increases in stiffness and material strength [37]. In a subsequent study (2020), the influence of different CLP concentrations (5%, 10%, and 15% by weight) on the dynamic mechanical properties of these composites was analysed [38]. The effect of CLP filler particle size on the thermal and mechanical properties of epoxy composites was also investigated [39].

In studies published in 2023, the potential use of lime peel as a novel bio-filler in thermoplastic composites was analysed, yielding favourable results, particularly increases in tensile and flexural moduli, thereby improving the material’s functional properties. The authors demonstrated that for a 10% filler addition, the tensile modulus increased by approximately 20.23% and the flexural modulus by approximately 14.33% compared to pure PP [40]. Meanwhile, Poikelispää and colleagues [41] investigated the dielectric and activation properties of polyacrylic rubber (ACM) modified with various plant-based fillers, such as potato starch, corn starch, garlic, and paprika. Mrówka and co-authors [42] evaluated the influence of organic waste derived from citrus peels—grapefruit, key lime, lemon, and orange—on the physical properties of silicone composites, confirming the positive effect of such bio-fillers on the structure and functionality of polymeric materials.

The results of previous studies clearly indicate that plant-based waste, particularly citrus fruit processing residues, constitutes a promising raw material for polymer composite technologies. Their utilisation enables the development of a new generation of biocomposites with improved functional properties, while simultaneously reducing the environmental impact.

Given the existing literature, it appears justified to continue research on the practical application of citrus waste as a bio-filler in polymer composites. The primary aim of this study is to develop a sustainable composite material for non-structural applications (e.g., casing elements, decorative panels) where high stiffness and surface hardness are prioritised over tensile strength. This approach balances the ‘green chemistry’ goal of utilising a significant volume of organic waste with the engineering requirement for functional durability. To achieve this, the study investigates the influence of varying weight fractions (up to 10 wt.%) of grapefruit, key lime, lemon, and orange peels on the mechanical performance and microstructure of polyester resin-based composites.

## 2. Materials and Methods

### 2.1. Materials

To address contemporary sustainability challenges and trends in the reuse of agro-industrial waste, the study focused on innovative thermoset composites reinforced with organic micro-fillers derived from citrus peel waste. AROPOL M 105 TB, a polyester resin (Ashland, Wilmington, DE, USA) with low styrene emission (UPR), was used as the polymer matrix. The crosslinking process was thermally initiated using 1 wt.% of Butanox M-50 methyl ethyl ketone peroxide (MEKP) (Akzo Nobel, 3800 AE Amersfoort, The Netherlands), which was handled under strict safety precautions due to its high reactivity. In research on thermoset composites (such as polyester and epoxy resins) with natural fibres, this matrix is commonly used, despite known challenges related to adhesion to hydrophilic natural fillers.

Food waste in the form of citrus fruit peels was used in this study, including grapefruit (*Citrus paradisi*), key lime (*Citrus aurantifolia*), lemon (*Citrus limon*), and orange (*Citrus sinensis*). This choice is consistent with the search for alternative, economically viable, and biodegradable fillers, rather than for the entire composite. To ensure reproducibility of results and minimise variability arising from environmental factors or fruit ripeness, all fruits were sourced from a single batch by a certified regional supplier (FUX Import-Export, Gliwice, Poland). Only fully ripe fruits with undamaged peels were selected for processing.

The citrus peels (grapefruit, key lime, lemon, and orange) were first dried in a KC-65 laboratory dryer (Premed, Warsaw, Poland) at 110 °C for 3 h. Subsequently, the dried peels were ground using a disc vibration mill (Testchem, Pszów/Radlin, Poland). The process parameters applied for each type of citrus peel included a mill load of 10 g/min and a grinding time of 1 min.

Grain composition analysis was performed using a Fritsch ANALYSETTE laboratory sieve shaker (Fritsch, Idar-Oberstein, Germany) equipped with a set of sieves with mesh sizes of 0.32 mm, 0.22 mm, 0.18 mm, 0.125 mm, 0.065 mm, and 0.03 mm. The duration of the sieve analysis for each powder tested was 60 min.

Microscopic analysis was conducted using a high-resolution scanning electron microscope SUPRA 35 (Carl Zeiss AG, Birkerod, Germany). Prior to microscopic examination, the powders were sputtered onto a carbon strip using an SCD050 BAL-TEC sputter coater (Capovani Brothers Inc., Scotia, NY, USA).

### 2.2. Methods

#### 2.2.1. Composites Preparation

Composites were manufactured using gravity casting. While gravity casting is a cost-effective method for preliminary screening, it entails limitations due to potential air entrapment relative to vacuum-assisted techniques. A series of samples was prepared with filler concentrations of 2.5%, 5%, and 10wt.%. This concentration range is typical for preliminary studies on polymer composites. The matrix-to-initiator ratio was 100:1 wt.

The fillers were mechanically mixed with the resin using a laboratory stirrer at 500 rpm for 10 min to promote dispersion, although inherent limitations regarding air entrapment in high-viscosity blends are acknowledged. Subsequently, the initiator was added, and the mixture was manually stirred for an additional 2 min. Prior to casting, the mixture was degassed by waiting for 15 min. The key stage of curing was made under a controlled thermal regime (instead of heating with a masonry reflector), consisting of primary curing at an elevated temperature (the primary curing was conducted at 50 °C for 6 h under controlled condition) followed by post-curing at ambient conditions (48 h), which is a standard procedure to achieve a high degree of crosslinking. The composite designations are shown in Table 1.

#### 2.2.2. Material’s Properties

Mechanical tests were conducted in accordance with EN ISO 527-1 [44] on five samples from each test group. The tests were conducted on the MTS Insight 2 (MTS Systems Corporation, Eden Prairie, MN, USA) testing machine equipped with an extensometer. The stretching speed of the samples was 50 mm/min. During the tensile test, Peak Load, Peak Stress, and Young’s modulus were measured. The results are presented as the arithmetic mean of five measurements, along with the standard deviation across all tested materials. The Shore D hardness test was performed in accordance with PN-EN ISO 868:2005 [45]. A Sauter TI-D (Sauter Automatyka Sp. z o.o., Warsaw, Poland) hardness tester was used for the tests. For each tested material, five measurements were made.

## 3. Results

### 3.1. Characterisation of Fillers

The particle size analysis of the comminuted grapefruit, key lime, lemon, and orange peels is presented in Table 2.

Particle size distribution analysis of grapefruit, lime, lemon, and orange samples revealed distinct in fragmentation patterns among the fruits studied. Based on the raw data, three main size fractions were identified as follows: fine (<0.125 mm), medium (0.125–0.18 mm), and coarse (>0.18 mm). A distinct similarity was observed in the grain composition of grapefruit and lemon, both of which are classified as coarse-grained materials. For these fruits, more than 90 wt.% of the grains were larger than 0.125 mm. Specifically, the dominant fraction was retained on the 0.18 mm mesh sieve (corresponding to the 0.18–0.22 mm range), accounting for 45.53 wt.% of grapefruit samples and 50.85 wt.% of lemon samples. This indicates a resistance to micronisation during processing for these species.

In contrast, key lime and orange samples exhibited a prevalence of fine particles. A significant portion of the material fell into the fine fraction (<0.125 mm), constituting approximately 66.1 wt.% for key lime and 56.9 wt.% for orange. Within this fine fraction, the 0.065–0.125 mm range was particularly significant, containing about one-third of the total mass for both fruits (36.16 wt.% for lime and 32.76 wt.% for orange). Such a distribution suggests a brittle, porous microstructure typical of lignocellulosic fruit waste, resulting in a high specific surface area. In the specific context of this study, this fine particle size distribution appeared advantageous for use as a filler in polymer composites, as it promotes better dispersion within the matrix and may increase stiffness or the elastic modulus.

The estimated mean particle size, determined as a weighted average of the percentages, further emphasises these differences. Lime and orange exhibited significantly lower mean particle sizes (~0.10–0.12 mm) compared to grapefruit and lemon (~0.17–0.18 mm). The proportions of fine (<0.125 mm), medium (0.125–0.18 mm), and coarse (>0.18 mm) particles confirm these structural characteristics. For instance, the key lime ratio was approximately 66:17:16, indicating a fine-grained structure, whereas for grapefruit it was 7:40:53, demonstrating a clear predominance of medium and coarse sized particles.

Based on the above, it can be concluded that the comminuted key lime and orange peels contained a significantly higher number of grains smaller than 0.125 mm.

**Figure 1:**
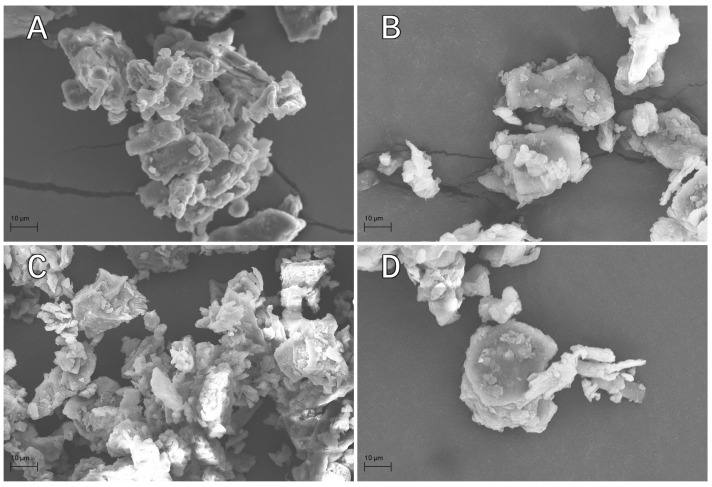
SEM images of the tested fillers: (**A**) grapefruit, (**B**) key lime, (**C**) lemon, (**D**) orange.

The SEM images of the analysed comminuted citrus peels (Figure 1) show that the particle shapes of the fillers are nearly identical for all citrus types, with the distinguishing feature being their size. Irregular and spherical grains were present (mainly for grains >75 μm), as well as flake-shaped grains (especially grains around 10 μm). It should be noted that the grains of the analysed citrus peels exhibited a characteristic layered structure. Qualitative observations suggest that further comminution could cause them to fracture along the cleavage plane, thereby producing more flake-shaped grains.

### 3.2. Mechanical Properties

**Figure 2:**
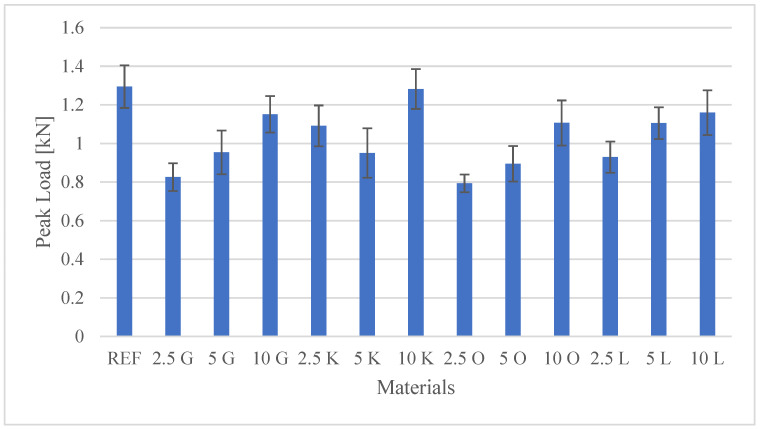
Peak Load values for the tested materials.

**Figure 3:**
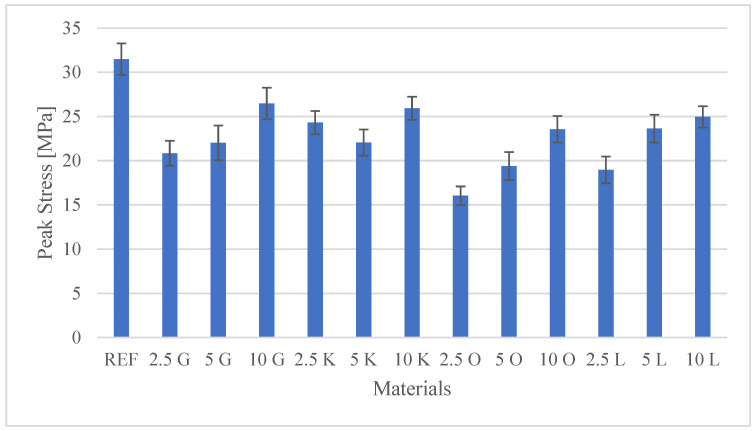
Peak Stress values for the tested materials.

**Figure 4:**
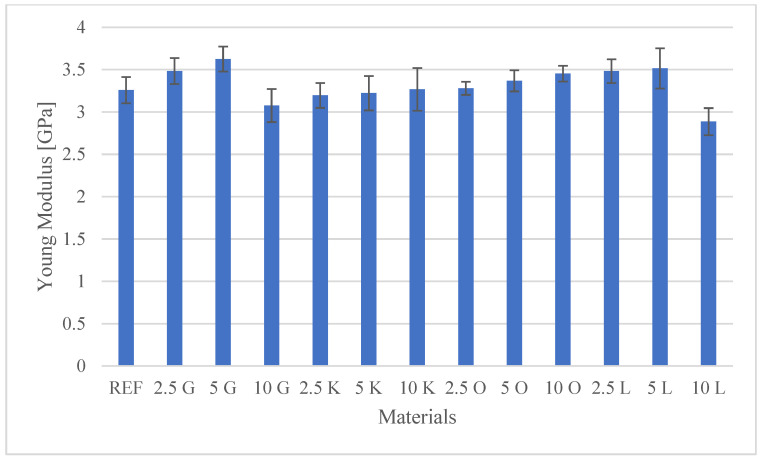
Young’s modulus values for the tested materials.

**Figure 5:**
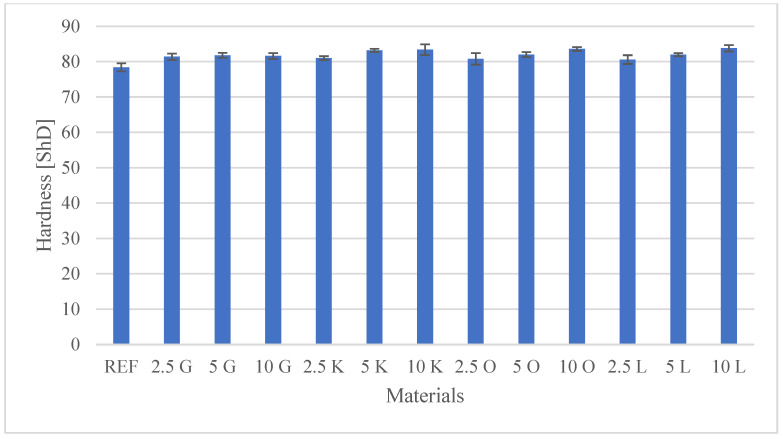
Shore D hardness characterisation of the tested materials.

The results of the investigations, encompassing both the tensile test and the hardness measurements, are presented in Table 3. Additionally, detailed mechanical characteristics are illustrated in the figures: Figure 2 shows Peak Load values, Figure 3 shows Peak Stress, Figure 4 shows Young’s modulus, and Figure 5 shows hardness test results.

Based on the static mechanical characterisation, the measurement of the maximum load (Peak Load) indicated that the incorporation of citrus peel microparticles as particulate fillers into the polyester resin (PR) matrix, a thermosetting binder, resulted in an overall lower ultimate tensile strength than the neat resin (REF), although a trend toward higher Peak Load was observed with increasing filler loading in the grapefruit, orange, and lemon samples. The neat polyester resin achieved a Peak Load of 1.29 kN, the highest recorded result among all tested materials, consistent with observations that untreated bio-waste fillers tend to decrease mechanical performance due to poor interfacial adhesion.

Detailed analysis of the effect of filler weight percentage (wt.%) revealed varying trends dependent on the citrus peel type:

1. Grapefruit (G), orange (O), and lemon (L): For these fillers, a progressive increase in maximum load was observed with increasing filler content, suggesting that higher concentrations may more effectively counteract the stiffening or weakening of the matrix. The lowest value recorded was 0.79 kN (2.5O) of approximately 40% compared to the neat polyester resin. The load increase was significant:
  - Grapefruit: from 0.83 kN (2.5G) to 1.15 kN (10G).
  - Orange: from 0.79 kN (2.5O) to 1.11 kN (10O).
  - Lemon: from 0.93 kN (2.5L) to 1.16 kN (10L).
2. Key lime (K): Composites reinforced with key lime filler exhibited an unusual concentration-dependent trend: 1.09 kN (2.5K) → 0.95 kN (5K) → 1.28 kN (10K). Notably, the composite with the highest filler loading, 10K, achieved a significant performance enhancement (1.28 kN), closely approximating the neat polyester resin (1.29 kN) and establishing itself as the highest Peak Load among all developed biocomposites. This result suggests that 10 wt.% may represent an optimal loading for the key lime filler in this specific matrix, mitigating common issues associated with high filler content, such as void formation and particle agglomeration. This finding supports the potential use of key lime peel particles as a sustainable filler resource, thereby reducing petroleum-based polyester consumption.

The tensile mechanical performance, specifically the Peak Stress (ultimate tensile strength), was evaluated to assess the effect of incorporating citrus peel waste into the polyester resin (PR) matrix. The neat polyester resin (REF) exhibited the highest Peak Stress at 31.48 MPa. This superior performance of the pristine matrix is expected, as the inclusion of untreated natural fillers often degrades the mechanical properties of polymer composites. The reduction in strength across all composite formulations is primarily attributable to poor interfacial adhesion between the hydrophilic lignocellulosic components of citrus peels and the hydrophobic PR matrix.

A detailed examination of the effect of filler loading (wt.%) on Peak Stress revealed specific trends across the different citrus peel types:

1. Grapefruit (G), orange (O), and lemon (L): These three types of particulate fillers showed a consistent trend where Peak Stress increased progressively with increasing filler content (from 2.5 wt.% to 10 wt.%) within their respective series.
  - For grapefruit (G) composites, the stress increased from 20.84 MPa (2.5G) to 26.46 MPa (10G).
  - The orange (O) composite at 2.5 wt.% (2.5O) recorded the lowest Peak Stress (16.04 MPa) among all tested materials, representing approximately a 50% reduction relative to the neat resin (REF). This finding highlights a severe weakening effect at this low loading. Increasing the orange content increased the stress to 23.55 MPa (10O).
  - Lemon (L) composites showed an improvement from 18.98 MPa (2.5L) to 24.96 MPa (10L). The values obtained for 5L (23.64 MPa) and 10L (24.96 MPa) are highly comparable, suggesting that the system may reach saturation in load-bearing capacity around 5 wt.%.
2. The key lime (K) filler exhibited a non-monotonic response, indicating complex interactions influenced by concentration. The stress was 24.32 MPa (2.5K), followed by an unusual drop to 22.04 MPa (5K), before recovering significantly to 25.93 MPa (10K). Such behaviour may result from dispersion challenges or particle agglomeration at intermediate filler concentrations.

Young’s modulus (E), which serves as a critical parameter for the material’s tensile stiffness, was determined for the neat polyester resin (REF) and the citrus peel-reinforced composites. The REF sample exhibited a Young’s modulus of 3.26 GPa. The incorporation of microparticulate citrus waste fillers produced heterogeneous effects on the matrix stiffness. In the case of grapefruit (G) and lemon (L), and across the entire concentration range, an initial increase in Young’s modulus was observed for orange (O), a desirable effect often attributed to more effective load transfer by the stiff filler phase. The highest Young’s modulus value (3.63 GPa) was observed for the composite with 5 wt.% grapefruit (5G). This value exceeded the modulus of the neat resin by more than 10%, suggesting that optimal filler loading had been achieved for stiffness and strong interfacial bonding. This phenomenon contrasts with the general tendency towards degradation of mechanical properties observed in some composites with CLP fillers, e.g., epoxy matrices, where the elastic modulus can decrease even at 5% loading. The orange (O) composites showed a consistent, progressive increase in modulus with increasing filler content, reaching 3.45 GPa at 10 wt.% (10O). An initial increase in stiffness was also evident for lower lemon concentrations: 3.48 GPa (2.5L) and 3.51 GPa (5L). The effect of key lime peel (K) was relatively neutral. The Young’s modulus values for concentrations of 2.5 wt.% (3.19 GPa), 5 wt.% (3.22 GPa), and 10 wt.% (3.27 GPa) remained close to the reference value (3.26 GPa), indicating that this filler did not introduce significant reinforcement, but did not lead to significant stiffening or weakening either. Among the tested materials, the 10L composite exhibited the lowest modulus (2.89 GPa), approximately 10% lower than the REF value. The 10G sample (3.08 GPa) exhibited a performance decrement compared to both the optimal 5G composite and the REF matrix.

Shore D hardness is an essential indicator of a material’s resistance to permanent indentation and is fundamentally related to the material’s viscoelastic properties and modulus of elasticity. The neat polyester resin (REF) established a baseline hardness of 78.4 ShD. Consistent with the scientific literature on the incorporation of rigid fillers, all developed biocomposites exhibited increased hardness relative to the neat resin. This enhancement is typically attributed to the inherent rigidity of the lignocellulosic citrus peel particles, which act as a stiff phase resisting surface penetration.

Key lime (K), orange (O), and lemon (L): for these fillers, a progressive (monotonic) increase in hardness was observed with increasing filler mass fraction. This is a typical response to increasing the concentration of the rigid phase in the matrix. For key lime (K), hardness rose from 81.0 ShD (2.5K) to 83.4 ShD (10K). For orange (O), the value increased from 80.8 ShD (2.5O) to 83.6 ShD (10O). The highest hardness value (83.8 ShD) was achieved by the 10L composite (Lemon, 10 wt.%). This result indicates approximately 7% increase relative to the neat resin (REF). In the composites with lemon (L), the increase was clear, reaching 80.6 ShD (2.5L) and 82.0 ShD (5L).

The grapefruit (G) filled composites displayed nearly uniform hardness values across the entire concentration range (2.5G:81.4 ShD, 5G:81.8 ShD, 10G:81.6 ShD). This stability suggests that even the low concentration (2.5 wt.%) was sufficient to achieve the maximum stiffening effect attributable to the grapefruit microparticles, indicating that the surface resistance did not benefit significantly from further increases in concentration.

In general, the increase in Shore D hardness across all developed biocomposites is desirable and may indicate increased resistance to abrasive wear.

## 4. Discussion

The research focused on the secondary use of citrus waste—which accounts for approximately 50% of total fruit weight and is a significant environmental concern—by integrating it as a micro-filler into a polyester resin (PR) matrix [37,46]. The use of waste materials, such as *Citrus limetta* peel (CLP) and other agricultural wastes (e.g., coconut husks, rice husks, hemp), is considered an economical, environmentally friendly, and practical approach to developing polymer composites [34,35,36,37,38,39].

To address the issue of citrus waste accumulation, this study investigates the viability of incorporating grapefruit, key lime, lemon, and orange peels into an unsaturated polyester resin (UPR) matrix. Polyester resins are widely used in industrial applications but often suffer from brittleness and high polymerisation shrinkage. The addition of organic bio-fillers aims to modify these properties while reducing the consumption of the petrochemical binder. The prepared composites were subjected to tensile and hardness tests to evaluate their suitability for semi-structural applications.

The incorporation of dried and ground citrus peels into the polyester matrix resulted in distinct changes in mechanical performance, which can be correlated with the filler characteristics presented in Section 3.1. The initial tensile strength (Peak Stress) of the neat polyester resin (REF) was 31.48 MPa. The introduction of untreated fillers resulted in a general decrease in tensile strength across most samples. This reduction is a typical phenomenon observed in composites reinforced with untreated hydrophilic natural fillers. The primary mechanism for strength reduction is poor interfacial adhesion between the polar components of the citrus peels, which are rich in pectin, cellulose, and hemicellulose; the chemical composition of these components is well-documented in previous studies and confirms their hydrophilic nature [47,48].

This incompatibility leads to ineffective stress transfer and the formation of micro-voids at the interface, which act as stress concentrators. It is important to acknowledge that the void content was not quantitatively assessed in this study (e.g., through density measurements), and no micro-structural analysis of the composite fracture surfaces was performed. Therefore, the presence of these defects (Figure 1) and the dispersion state are inferred from the filler granulometry and mechanical response, rather than from direct microscopic observation of the interface. The magnitude of this effect varied with filler type and concentration, which can be linked to the particle size distribution (Table 2) and morphology (Figure 1). The key lime (10K) composite demonstrated a unique performance, achieving a Peak Load (1.28 kN) almost identical to that of the neat resin (1.29 kN). As shown in the granulometric analysis, key lime and orange fillers were characterised by a significantly finer fraction (approximately 68–77% of particles smaller than 0.125 mm), whereas grapefruit and lemon were coarser (dominated by the 0.125–0.22 mm fraction). We hypothesise that key lime’s finer particle size allows for better packing density at higher loadings (10 wt.%), which could potentially mitigate the volume of resin defects despite the poor chemical affinity. This is consistent with the quantitative data in Table 2, which shows that key lime had the highest content (66.1%) in the fine fraction (<0.125 mm), thereby directly contributing to the recovery of load-bearing capacity relative to coarser fillers. This suggests that 10 wt.% represents an optimal loading for the finer key lime filler, maximising the load-bearing capacity before agglomeration becomes critical.

Although this study focused primarily on mechanical and morphological characterisation, previous literature data indicate that citrus peel biomass remains thermally stable up to approximately 200 °C, which is well above the curing temperature of the polyester resin used in this study. Based on reported literature values for lignocellulosic waste, thermal degradation is not expected to be a technological barrier [37]. In summary, it is reasonable to assume thermal stability of citrus fillers under standard processing conditions and to focus research on their mechanical characterisation, a justified scientific strategy supported by the literature [49,50].

In terms of stiffness, the grapefruit (5G) composite exhibited the highest Young’s modulus of 3.63 GPa, an increase of over 10% compared to the reference. The SEM images (Figure 1) revealed that the filler particles possess a layered, irregular structure with some flake-like geometry. The coarser nature of the grapefruit particles (dominant fraction 0.125–0.18 mm) likely contributed to a more effective stiffening effect at lower concentrations (5 wt.%) compared to the finer fillers, as larger aspect ratio particles (inferred from qualitative SEM analysis) can be more efficient in restraining the mobility of polymer chains. Conversely, the 10L (lemon) composite exhibited the lowest modulus (2.89 GPa). Although lemon particles are similarly coarse to grapefruit, the high loading of 10 wt.% likely led to particle-to-particle contact and agglomeration, which, when combined with weak interfacial adhesion, disrupted the matrix continuity and reduced stiffness.

A key positive finding was the universal improvement in Shore D hardness across all biocomposites relative to the neat resin (78.4 ShD). The maximum hardness was achieved by the 10L composite (83.8 ShD), representing an approximately 7% increase. The SEM analysis confirmed the presence of rigid, irregular lignocellulosic particles. The monotonic increase in hardness with filler content (particularly for key lime, orange, and lemon) confirms that the rigid filler phase effectively resists surface indentation. While hardness is not a direct measure of wear resistance, this improvement suggests that the developed biocomposites may offer enhanced resistance to surface scratching and deformation in non-structural applications, such as decorative panels or casing elements.

To provide a comprehensive assessment of reinforcement efficiency, a quantitative analysis of relative property changes relative to the reference material was performed (Table 4).

Table 4 quantifies the trade-off between strength and stiffness. While Peak Stress decreased across all samples (reaching a maximum decrease of −49.1% for 2.5O), Shore D hardness increased across all samples (up to +6.9% for 10L). Notably, the 5G sample exhibits the largest stiffness gain (+11.4%), whereas the 10K sample showed a negligible loss in load-bearing capacity (−0.8% in Peak Load) relative to the reference material. No direct correlation was found between modulus and tensile strength; filler incorporation increased surface stiffness and bulk rigidity but simultaneously reduced the ultimate load-bearing capacity due to the dominance of interfacial defects over the reinforcement effect.

Furthermore, descriptive statistics of the entire dataset indicate that while Peak Stress showed high variability (Standard Deviation = 3.90 MPa), reflecting sensitivity to local defects and interfacial adhesion, Young’s Modulus and Hardness exhibited high stability (SD = 0.20 GPa and 1.49 ShD, respectively). This confirms that the stiffening effect of the bio-filler is consistent and less sensitive to processing variations than the tensile strength.

The non-monotonic tensile behaviour observed for the key lime series (where 5K showed lower performance than 2.5K and 10K) can be attributed to the interplay between particle dispersion and agglomeration. For fine powders such as key lime, intermediate concentrations may exhibit uneven dispersion (agglomeration) that is insufficient to form a percolating network but large enough to create critical defects. At 10 wt.%, the volume fraction is sufficient to distribute the load more evenly, as evidenced by the recovery in Peak Load.

Regarding long-term stability, we assume that the polyester matrix protects the bio-filler from environmental factors. However, the literature suggests that natural fillers can degrade over time due to moisture absorption and biodegradation. Prolonged exposure to humidity may lead to moisture absorption by the filler, potentially causing dimensional changes or degradation of the filler–matrix interface, which could gradually alter the mechanical performance of the entire composite. Therefore, while the material appears suitable for the proposed non-structural applications, future long-term ageing studies are essential to validate the service life of these materials.

It must be noted that while the drying process (110 °C for 3 h) minimised moisture content, the lack of chemical surface modification remains a limiting factor for tensile strength. Future work should focus on chemical treatments, such as alkalisation or silanisation, as well as thermal ageing tests, to reduce the hydrophilicity of the fillers and improve the filler–matrix interaction, potentially unlocking structural applications for these materials.

## 5. Conclusions

The findings confirm the efficacy of utilising citrus peel waste as micro-fillers to reduce the reliance on petrochemical polymers, highlighting the material’s potential to contribute to circular economy (CE) strategies.

Granulometric analysis and SEM imaging revealed distinct morphological differences between the fillers. Key lime and orange peels were characterised by a dominant fine fraction (mostly <0.125 mm), while grapefruit and lemon fillers contained a significant portion of coarser particles (0.125–0.22 mm). All fillers exhibited a layered, irregular structure with flake-like particles, which is critical for their interaction with the matrix.

The incorporation of untreated waste into the polyester resin (PR) matrix resulted in a general decrease in ultimate tensile strength (Peak Stress REF: 31.48 MPa). However, the analysis demonstrated a significant improvement in key functional properties at optimal filler concentrations, thereby validating the use of these materials in non-structural applications where cost reduction, waste management, and surface durability are prioritised. The stiff filler phase proved effective at stiffening the matrix, particularly at low loadings. The highest Young’s modulus (3.63 GPa) was recorded for the 5G (5 wt.% grapefruit) composite, exceeding the stiffness of the neat resin (REF: 3.26 GPa) by more than 10%. Consistent with the incorporation of rigid particulate fillers, all developed biocomposites exhibited universal improvements in Shore D hardness. The maximum hardness (83.8 ShD) was achieved by the 10L (10 wt.% lemon) composite, representing an approximately 7% increase over the neat resin (REF: 78.4 ShD). Despite the overall strength reduction, the 10K (10 wt.% key lime) composite demonstrated a Peak Load (1.28 kN) almost identical to that of the neat resin (1.29 kN), suggesting that this specific concentration may constitute an optimal loading for key lime in terms of maximum load capacity.

However, because this study was limited to static tensile and hardness tests, the results should be considered a preliminary screening. To fully qualify these materials for specific industrial applications, additional mechanical characterisation will be required, including flexural properties, wear resistance, impact strength, and dynamic mechanical analysis (DMA).

To overcome the performance limitations, primarily the poor interfacial adhesion between the naturally hydrophilic citrus peel components (rich in cellulose and pectin) and the hydrophobic UPR matrix, future work is strongly recommended to incorporate chemical surface modification treatments of the fillers, such as alkaline (NaOH) treatment. Such treatments are known to remove non-cellulosic components (hemicellulose and lignin), improving fibre wetting and enhancing the overall mechanical and thermal properties of natural fibre composite.

## Figures and Tables

**Table 1:** Composition of samples [43].

Material	Filler	Filler Content (%)
REF	-	0
2.5G	grapefruit	2.5
5G	grapefruit	5
10G	grapefruit	10
2.5K	key lime	2.5
5K	key lime	5
10K	key lime	10
2.5L	lemon	2.5
5L	lemon	5
10L	lemon	10
2.5O	orange	2.5
5O	orange	5
10O	orange	10

**Table 2:** Particle size distribution of ground citrus peels.

Size [mm]	Grapefruit	Key lime	Lemon	Orange
>0.32	4.08	3.81	0.77	0.55
0.32–0.22	2.75	1.52	1.35	0.52
0.22–0.18	45.53	11.07	50.85	4.67
0.18–0.16	19.25	5.64	14.04	10.97
0.16–0.125	20.82	11.21	25.35	26.4
0.125–0.065	5.69	36.16	3.27	32.76
0.065–0.03	0.97	20.97	3.64	18.61
0.03–0.02	0.19	8.98	0.74	5.52
<0.02	0	0	0	0

**Table 3:** Tensile and Shore D hardness test results of the investigated materials.

	Peak Load [kN]	Peak Stress [MPa]	Young’s Modulus [GPa]	Hardness [ShD]
REF	1.29	31.48	3.26	78.4
2.5G	0.83	20.84	3.49	81.4
5G	0.95	22.02	3.63	81.8
10G	1.15	26.46	3.08	81.6
2.5K	1.09	24.32	3.19	81
5K	0.95	22.04	3.22	83.2
10K	1.28	25.93	3.27	83.4
2.5O	0.79	16.04	3.28	80.8
5O	0.89	19.4	3.37	82
10O	1.11	23.55	3.45	83.6
2.5L	0.93	18.98	3.48	80.6
5L	1.11	23.64	3.51	82
10L	1.16	24.96	2.89	83.8

**Table 4:** Relative percentage change () of mechanical properties compared to the reference material (REF).

	Young’s Modulus [GPa]	Δ Young’s Modulus [%]	Hardness [ShD]	Δ Hardness [%]
REF	3.26	0.0	78.4	0.0
2.5G	3.49	+7.1	81.4	+3.8
5G	3.63	+11.4	81.8	+4.3
10G	3.08	−5.5	81.6	+4.1
2.5K	3.19	−2.2	81	+3.3
5K	3.22	−1.2	83.2	+6.1
10K	3.27	+0.3	83.4	+6.4
2.5O	3.28	+0.6	80.8	+3.1
5O	3.37	+3.4	82	+4.6
10O	3.45	+5.8	83.6	+6.6
2.5L	3.48	+6.7	80.6	+2.8
5L	3.51	+7.7	82	+4.6
10L	2.89	−11.4	83.8	+6.9

## Data Availability

The original contributions presented in this study are included in the article. Further inquiries can be directed to the corresponding author.
